# The Metabolic Prospective and Redox Regulation of Macrophage Polarization

**DOI:** 10.4172/2155-9899.1000371

**Published:** 2015-11-30

**Authors:** Chao He, A Brent Carter

**Affiliations:** 1 Department of Medicine, University of Alabama at Birmingham, Alabama, USA; 2 Division of Pulmonary, Allergy, and Critical Care Medicine, University of Alabama at Birmingham, Alabama, USA; 3 Birmingham VAMC, Birmingham, Alabama, USA

**Keywords:** Macrophage, Macrophage polarization, Alternative activation, Pulmonary fibrosis, Mitochondria, Reactive oxygen species, Fatty acid oxidation

## Abstract

Macrophage plasticity is an important feature of these innate immune cells. Macrophage phenotypes are divided into two categories, the classically activated macrophages (CAM, M1 phenotype) and the alternatively activated macrophages (AAM, M2 phenotype). M1 macrophages are commonly associated with the generation of proinflammatory cytokines, whereas M2 macrophages are anti-inflammatory and often associated with tumor progression and fibrosis development. Macrophages produce high levels of reactive oxygen species (ROS). Recent evidence suggests ROS can potentially regulate macrophage phenotype. In addition, macrophages phenotypes are closely related to their metabolic patterns, particularly fatty acid/cholesterol metabolism. In this review, we briefly summarize recent advances in macrophage polarization with special attention to their relevance to specific disease conditions and metabolic regulation of polarization. Understanding these metabolic switches can facilitate the development of targeted therapies for various diseases.

## Origins of Macrophages

Macrophages are innate immune cells of the mononuclear phagocyte system that play an important role in the cross-talk between the innate and adaptive immunity [1,2]. The function of macrophages varies significantly with regard to tissue specificity, such as the alveolar macrophages, the adipose tissue macrophages, Kupffer cells in the liver, and microglia cells in the central nerve system. Based on the expression of F4/80, murine tissue macrophages can trace their origin back to two sources. Macrophages that are derived within bone marrow usually express low F4/80, whereas macrophages that originate from the embryonal yolk sac typically express high level of F4/80 and are capable of proliferating *in situ*, a scenario seen in the radiation-induced chimeras and the bone marrow transplant mice [3–5]. To further delineate the circulating monocyte derived macrophages, studies using specific surface markers, lymphocyte antigen 6C (Ly6C), C-C chemokine receptor type 2 (CCR2), and CX3C chemokine receptor 1 (CX3CR1), two sets of monocytes are identified: the Ly6C-^high^ and Ly6C-^low^ monocytes [6]. The Ly6C^high^ monocytes are inflammatory monocytes, which have high expression of CCR2 and a low level of CX3CR1. They are short-lived and rapidly recruited to the site of inflammation during the acute infectious process. The Ly6C^low^ monocytes do not express CCR2 but have high level of CX3CR1. They usually do not migrate immediately to the site of infection due to their low expression of CCR2. However, Ly6C^low^ monocytes are usually long-lived cells and play an important role in chronic processes, such as tumorigenesis and fibrotic remodeling.

## Macrophage Polarization

Macrophage polarization is a process through which macrophages obtain different phenotypes. The phenotype of a macrophage is closely related to the microenvironment in which they reside, as macrophages are able to switch phenotypes constantly both *in vivo* and *in vitro* [7,8]. In an analogy to the T-helper-cell nomenclature, where Th1 cells are associated with the response against bacteria or viruses, and Th2 cells are associated with the response to parasitic infection and tissue remodeling, macrophages can be denoted as M1 and M2 macrophages. M1 macrophages (or classically activated macrophages, CAMs) are pro-inflammatory and have potent microbicidal and tumoricidal activity, whereas the M2 macrophages (or alternatively activated macrophages, AAMs) are involved in tumor progression and tissue remodeling, including fibrosis [9,10].

**Figure 1:**
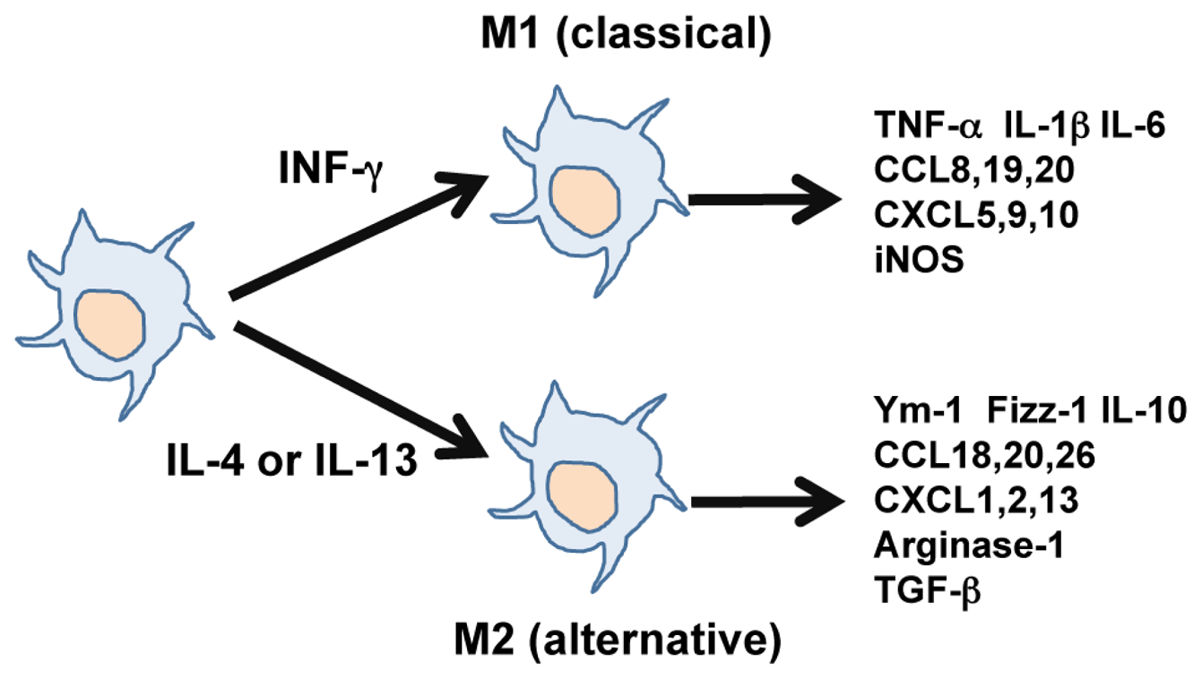
General concepts of macrophage polarization and properties of M1 and M2 macrophages. INF-γ induces M1 (classical) macrophage polarization whereas IL-4 and/or IL-13 induce M2 (alternative) macrophage polarization.

Classical macrophage activation requires priming with IFN-γ, the canonical cytokine generated by Th1 cells, and activation of the downstream transcription factors, such as signal transducer and activator of transcription 1 (STAT1), nuclear factor-kappa lightchain-enhancer of activated-B cells (NF-κB), and interferon regulatory factor 5 (IRF-5). These M1 macrophages express inflammatory genes, including TNF-α, IL-1β, and IL-6. Alternatively activated macrophages are usually activated by Th2 cytokines, IL-4 and/or IL-13. The wide range of immunosuppressive cytokines and growth factors alternatively activated macrophages produce, such IL-10, IL-1ra (IL-1 receptor antagonist), and transforming growth factor-β (TGF-β), are closely related to their ability to attenuate inflammation and promote extracellular tissue remodeling. Transcription factors involved in M2 polarization include STAT3, STAT6, IRF-4, and peroxisome proliferator-activated receptor (PPAR)-γ (Figure 1). Differential metabolism of L-arginine is characteristic of M1 and M2 macrophages. L-arginine is metabolized by iNOS to generate nitric oxide (NO) in M1 macrophages and by arginase-1 in M2 macrophages to augment the production of polyamines and L-proline, which are essential substrates for collagen synthesis [11,12].

The origin of macrophages also plays a critical role in determining macrophage phenotype. *L. sigmodontis* infection induces M2 macrophage proliferation *in situ*, rather than by the recruitment and differentiation of circulating monocytes [13]. In contrast, in a LPS-induced COPD model, using MRI tracking of nanoparticles-labeled *ex vivo*, prepolarized bone marrow-derived macrophages, both M1 and M2 are recruited to the sites of inflammation in the lung at similar level [14].

## Macrophage polarization and human diseases

The classically activated M1 macrophages boast the basic macrophage function as implied by the name given by Elie Metchnikoff in 1887. They are the dominant cells in acute infection, participating in bacteria/pathogen clearance and antigen presenting by their effective phagocytic ability. They also have compelling tumoricidal activity. M2 macrophages are actively involved in many processes associated with parasitic infection, immune tolerance, wound healing, and tumorigenesis. The function of M1 and M2 macrophages are detailed below with a particular focus of M2 macrophage and human diseases.

### Inflammation, infection, and sepsis

The generation and role of alternatively activated macrophages (AAMs) has been studied extensively in helminth-related diseases [15–17]. After *N. brasiliensis* subcutaneous inoculation, their larvae travel to the lung and trigger a potent M2 polarization in alveolar macrophages [15]. Helminth infection not only initiates M2 polarization, but also is also capable of subverting the M1 polarization as shown in *Francisella tularensis* infection [18]. In an animal model of schistosomiasis, conditional macrophage/neutrophil IL-4 receptor alpha-deficient mice (LysM*^Cre^*-IL-4Rα ^(−/flox)^) show a predominant M1 polarization and more severe infection with 100% mortality [19]. Endotoxin or lipopolysaccharide (LPS) tolerance is the reduced responsiveness to LPS stimulus after repeated exposure. It is a common scenario in patients with persistent sepsis, especially in intensive care settings [20]. TNF-α production was significantly elevated in monocytes treated with one dose of LPS. However, if these cells were pre-challenged with the same dose of LPS 24 h before the second dose, the level of TNF-α production was greatly reduced [21]. Peripheral blood monocytes and macrophages from these patients often display features resembling alternative activation of monocytes, including reduced production of pro-inflammatory mediators and expression of genes involved in tissue remodeling [21,22]. Similarly, peripheral monocytes collected from septic patients have higher level of T17 and Treg cell populations with elevated CD206 and CD163 expression, suggesting LPS-tolerance and M2 polarization [23].

### Wound and tissue remodeling

Wound macrophages are known to undergo alternative activation [24]. Delayed healing occurs in mice with dysfunctional M2 macrophages or deficiency of signature M2 gene expression, such as *arg1* [25]. Arginase-1 is pertinent to fibrosis development as it metabolizes arginine to generate L-ornithine, which will be utilized by ornithine decarboxylase to generate L-proline and polyamines. While induction of arginase-1 by IL-4 and/or IL-13 is commonly believed to contribute to collagen deposition and fibrosis development [26,27], reports suggest that up-regulation of arginase-1 in macrophages actually inhibits fibrosis development as they compete with fibroblasts for arginine as the substrate for L-ornithine synthesis and by inhibiting Th2 cytokine production, particularly IL-13 [28]. Both IL-4 and IL-13 receptors have been shown to be essential for fibrosis development in *S. mansoni* granuloma formation [29]. Alternative activation of macrophages is the predominant macrophage phenotype in tissue samples from patients with chronic pancreatitis, and mice lacking IL-4Rα have less M2 macrophages and are protected from developing fibrotic changes after ceruletide injection [30]. By using an IL-4/IL-13 blocking peptide, similar anti-fibrotic effects can be achieved via inhibition of M2 polarization [30].

### Cardiovascular diseases

The exact mechanism of how different macrophage phenotypes influence myocardial remodeling remains largely unknown. M2 macrophages have been shown to be crucial for post-myocardial infarction remodeling as IL-13^−/−^ mice have significant worsening outcome in an infarction model compared to wild-type mice [31]. Another study showed that mineralocorticoid receptor knockout mice displayed a dominant M2 polarization pattern, and these mice are protected against cardiac hypertrophy, fibrosis, and vascular damage caused by angiotensin II. Additionally, aldosterone can induce M1 polarization, while eplerenone, an aldosterone antagonist, inhibits M1 activation, underscoring the cardioprotective role of M2 macrophages [32].

### Pulmonary diseases

Alternatively activated macrophages are also implicated in various pulmonary disorders, including COPD, asthma, pulmonary hypertension, and pulmonary fibrosis. Plasma Chitinase-1, a signature M2 protein, has been used to quantify disease severity in COPD patients [33]. One study shows a remarkable example of a pathogenic role of IL-13 in chronic obstructive pulmonary disease (COPD) that underscores the effect of M2 macrophages. The macrophages upregulate IL-13Rα1 expression and become alternatively activated by an autocrine or paracrine mechanism [34], which leads to COPD progression. The role of different macrophage phenotypes in pulmonary hypertension remains undetermined. It is known that fibroblast-derived IL-6 polarizes alveolar macrophages into an M1 pattern and drives the development of pulmonary hypertension in a paracrine fashion, together with activation of signature M1 transcription factors, STAT3 and HIF-1α [35]. Others have found that macrophages acquire an M2 phenotype during hypoxia, and M2 macrophages lead to the proliferation of pulmonary artery smooth muscle cells. Blocking M2 polarization can potentially attenuate the progression of pulmonary hypertension by attenuating smooth muscle cell proliferation [36]. Additionally, M2 macrophages are known to be prevalent in the lungs of patients with idiopathic pulmonary fibrosis, sarcoidosis, systemic sclerosis, asbestos-induced pulmonary fibrosis, and gamma-herpes virus-induced pulmonary fibrosis [7,37,38]. Conversely, mice with predominant M1 macrophages are protected from developing asbestos-induced pulmonary fibrosis [7,39]. Similarly, in a bleomycin-induced pulmonary fibrosis model, both fibrosis and alternative activation of macrophages are prolonged in TNF-α^−/−^ mice. Intra-tracheal delivery of recombinant TNF-α can ameliorate established pulmonary fibrosis, partially *via* inducing Fas-mediated fibroblast apoptosis [40,41]. Moreover, CCL-18, a signature M2 chemokine, is known to induce lung fibroblast collagen production [42], highlighting the importance of crosstalk between macrophages and fibroblasts.

### Cancer

Tumor-associated macrophages (TAMs) have many properties of M2 macrophages, and they contribute to tumor local invasion through secreting proteinases, such as cathepsin [43]. GB111-NH_2_, an inhibitor of cathepsin, decreases expression of the classic M2 genes, *fizz1* and *jmjd3*, resulting in tumor regression [44]. TAMs also promote angiogenesis and tumor growth through VEGF, leading to chemo-resistance [45,46]. M2 macrophages promote tumorigenesis by increasing signature M2 markers, such as CCL-18 [47]. IL-13, along with its receptors IL-13Rα2, induces TGF-β expression and contributes to tumor development by inhibiting cytotoxic T cells [48]. In contrast, blockage IL-13Rα2 *via* siRNA reduces metastasis and promotes survival [49]. Exposing TAMs to the canonical Th1 cytokine, INF-γ, can reprogram TAMs to acquire M1 features and regain anti-tumor activity [50]. Similarly, targeting transcription factors crucial for TAM differentiation, such as STAT3, can also achieve tumoricidal function [51]. Molecular inhibitors targeting M2 macrophages, such as the pro-apoptotic peptide [52] and anti-VEGF antibody [53], are considered to be potential candidates for cancer treatment.

## Metabolic regulation of macrophage polarization

### Redox status regulates macrophage polarization

The role of oxidative stress in macrophage polarization is controversial. The development of granulomas from *S. mansoni* exposure is not impaired in IL-4-deficient mice [54,55], as other Th2 cytokines remain elevated. In addition, wound macrophages are known to undergo alternative activation despite a deficiency of Th2 cytokines in the wound environment, and the macrophage phenotype is sustained in mice lacking IL-4R. It is not clear from these studies what induced the alternative activation.

Oxidative stress has long been known to play an important role in the development and progression of pulmonary diseases. Pro-inflammatory M1 genes, such as *tnf-α*, *il-1β*, and *inos*, have all been shown to be regulated by redox proteins, including Cu,Zn-SOD [56–58]. *ym1 and fizz1*, two signature M2 genes, are elevated in ovalbumin-challenged asthmatic mice, and their expression can be attenuated by treatment with *N*-acetylcysteine, a thiol-reducing agent, linking M2 polarization to oxidative stress [59]. Previous studies have shown that increases in the oxidative metabolic environment fuels alternative activation of macrophages [60], while others show that M2 macrophages generate low levels of ROS [61]. The H_2_O_2_ gradient, generated by dual oxidases (DUOX) in wound epithelium of zebrafish larvae, is known to be the chemo-attractants for macrophage recruitment [62]. IL-4-stimulated M2 macrophages have an enhanced mitochondrial oxygen-consumption rate [63], and inhibition of mitochondrial respiration by oligomycin dramatically increased the mRNA expression level of pro-inflammatory genes, such as *il-6*, *tnf-α*, and *il-1β*, underscoring an important role of mitochondrial respiration in M2 polarization [64].

**Figure 2:**
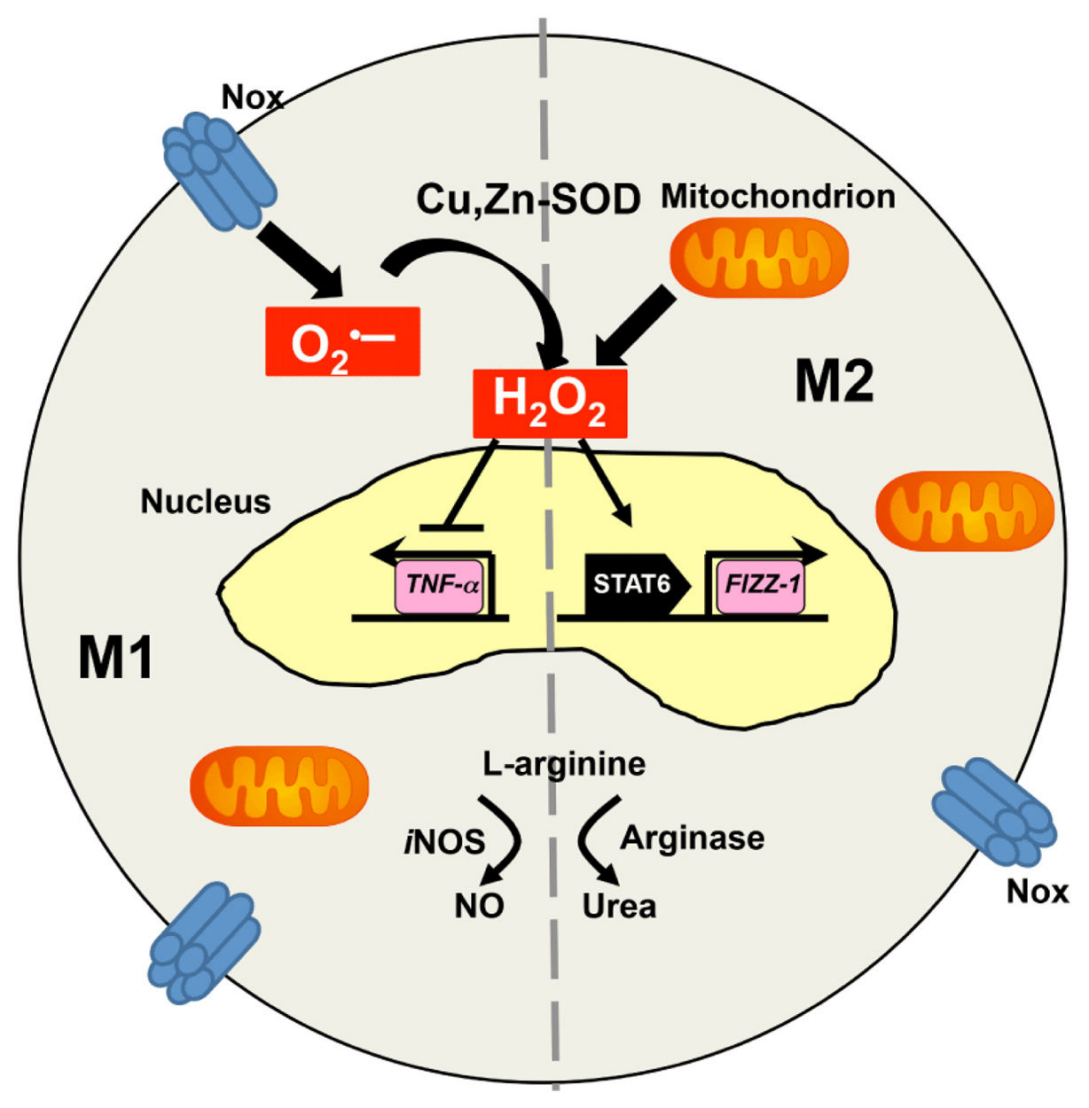
Redox regulation of macrophage polarization. Superoxide generated by either membrane-bound NADPH oxidase or mitochondrial electron transfer chain (ETC) will be converted to H_2_O_2_ by superoxide dismutase, which will inhibit M1 polarization and activate M2 polarization *via* STAT6. Revised from [7].

Data linking ROS to macrophage activation are emerging, but the exact role of ROS still requires further investigation. The loss of NADPH in a type I diabetes mouse model, superoxide-deficient bone marrow-derived macrophages had a marked reduction in proinflammatory M1 gene expression and showed increased M2 polarization, together with STAT6 activation [65]. Deficiency of nuclear-encoded protein NADH: ubiquinone oxidoreductase iron-sulfur protein 4 (Ndufs4), a critical component of mitochondrial complex I, is known to be related to impairment of oxidative phosphorylation [66]. Global *Ndufs4* loss causes systemic inflammation with a predominant M1 polarization [67]. At the same time, a metabolic shift from fatty acid oxidation (FAO) to glycolysis was observed in Ndufs4^−/−^ pups. Moreover, Ndufs4^−/−^ bone marrow macrophages have significantly higher superoxide levels, which can be attenuated by MitoTEMPO to further decrease pro-inflammatory gene expression. Conversely, circulating M2 macrophages accelerate the pathological progression of amyotropic lateral sclerosis (ALS), a disease characterized with aberrant Cu,Zn-SOD function and excessive H_2_O_2_ production [68]. Over-expression of Cu,Zn-SOD, the redox protein that catalyzes the generation of H_2_O_2_, polarizes macrophages to an M2 phenotype *via* activation of STAT6 with a cysteine residue (Cys_528_) serving as the redox switch [7]. Moreover, Cu,Zn-SOD-mediated macrophage polarization can be altered by modulating H_2_O_2_ generation. As previously mentioned, differential metabolism of L-arginine is characteristic of M1 and M2 macrophages. Overexpression of Cu,Zn-SOD leads to a reduction of *inos* gene expression and NO synthesis, while arginase-1 expression and urea generation is enhanced [7] (Figure 2). Acute chlorine gas exposure leads to oxidation of surfactant protein and augmentation of M2 genes, such as *arg1*, *fizz1*, and *ym1* [69]. Another study showed that alveolar macrophages exposed to ozone have elevated levels of both M1 and M2 genes [70]. Interestingly, one study has compared macrophage phenotype in two Nox2-deficient mouse models, gp91^phox−/−^ and p47^phox−/−^. Mice deficient in p47^phox−/−^ have a significant increase of M2 gene expression upon IL-4 stimulation and are protected from *Listeria monocytogenes* infection compared with gp91^phox−/−^ mice [71]. Explanations for the differences include that macrophage polarization is driven by specific reactive oxygen species (H_2_O_2_ vs O_2_^•−^), the different origin of ROS (membrane-bound NADPH oxidase, particularly Nox2 versus mitochondria), or the different tissue and intracellular distribution of NADPH oxidases or SODs.

Redox regulation in macrophage polarization is closely related to hypoxic conditions and hypoxia-inducible factors (HIFs) activation. In murine macrophages, the expression of hypoxia-inducible factors HIF-1α and HIF-2α appears to be dependent on respective inducers. M1-promoting factors induce the expression of HIF-1α, whereas IL-4 primarily induces HIF-2α that regulates M2 polarization [72]. HIF-1α^−/−^ macrophages exhibit diminished production of TNF-α and IL-6 in response to LPS/IFN-γ stimulation in a model of tumor spheroids [73].

Oxidative stress, particular the mitochondrial redox signal, is known to cause endoplasmic reticulum (ER) stress due to the proximal distance between mitochondria and ER [74]. Asbestos-treated macrophages, which show M2 polarization, have elevated ER stress with elevated level of binding immunoglobulin protein (BiP) and C/EBP homologous protein (CHOP) [75]. Induction of ER stress induces macrophage polarization from the M1 into the M2 phenotype leading to increased cholesterol deposition and enhanced foam cell formation [76]. MCP-1-induced protein (MCPIP), induced by either STAT6 or KLF-4, inhibits NF-κB in murine macrophages and instigates M2 polarization *via* induction of ER stress [77]. BiP and CHOP levels are elevated in THP-1 monocytes treated with ER-stress inducers, tunicamycin or thapsigargin, and the THP-1 cells undergo M2 polarization *via* the PPAR-γ pathway. Interestingly, M2 polarization could be reversed by treating with ER stress inhibitor 4-phenylbutyrate (PBA), emphasizing a potential therapeutic target [78].

### Metabolism of fatty acid/cholesterol regulates macrophage polarization

**Figure 3:**
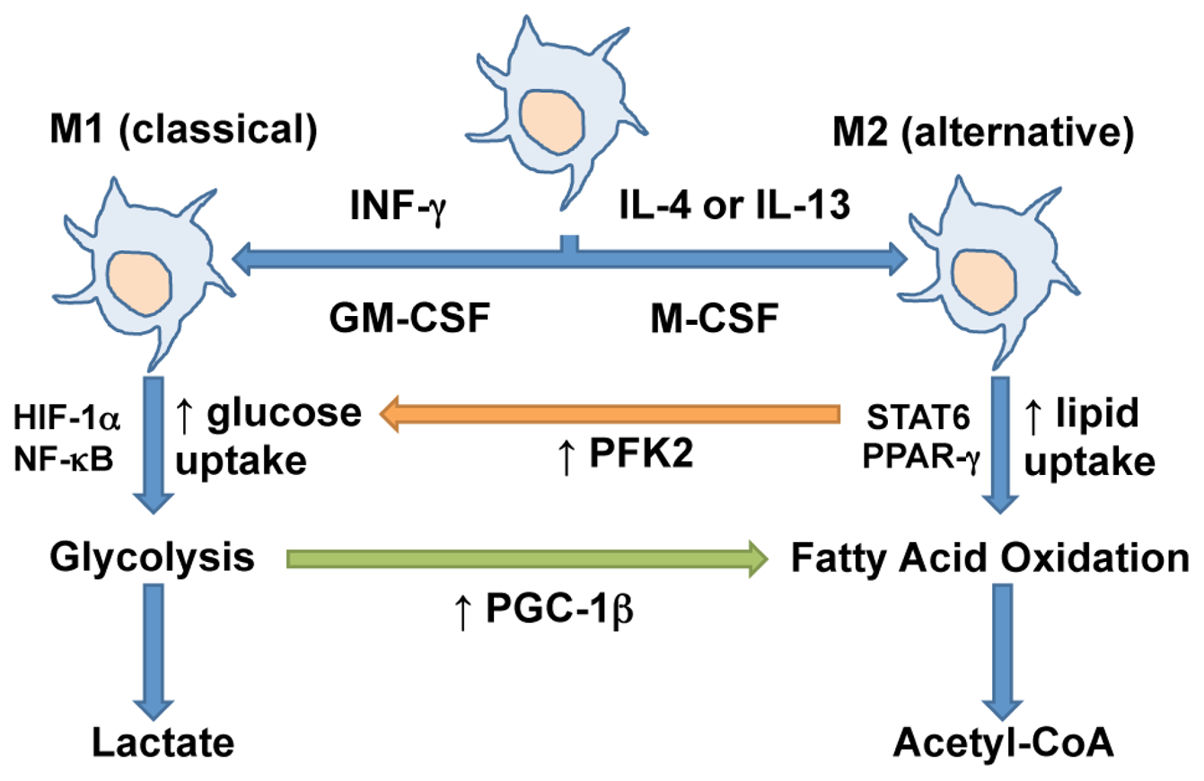
Metabolic regulation of macrophage polarization. M1 macrophages have increased uptake of glucose and augmented glycolysis, whereas M2 macrophages have increased uptake of lipid and augmented fatty acid oxidation. Specific cytokines and transcription factors regulate these pathways. Activation of PFK2 leads to M1 polarization while over-expressing PGC-1β leads to M2 polarization.

Prior data show that M2 polarization is dependent on fatty acid oxidation (FAO), whereas M1 macrophages rely on aerobic glycolysis [79]. The differences between the two metabolic pathways involve a switch in the expression of 6-phosphofructo-2-kinase/fructose-2,6-bisphostase (PFK2). M1 macrophages display a high expression of glycolytic enzymes and glycolysis-related metabolites. This shift toward aerobic glycolysis, known as the Warburg effect in cancer biology, rapidly provides immune cells with ATP and metabolic intermediates. In contrast, M2 macrophages have increased expression of genes encoding molecules in FAO and oxidative phosphorylation pathways [63]. Blocking oxidative metabolism not only selectively abrogates the ability of cells to undergo alternative activation but also potentiates the expression of M1 genes. Conversely, overexpressing PGC-1β, a key transcriptional proponent of oxidative metabolism, potentiates alternative activation and prevents classical activation by augmenting FAO [60] (Figure 3). Compared with M1 macrophages, which exert their functions over short time periods, M2 macrophages are engaged in long-term cellular activities, and the relative efficiency of FAO versus that of glycolysis is well suited to meet the metabolic requirements of their roles [80]. M2 macrophages have been shown to have longer survival compared to their M1 counterparts [63], and FAO is known to support cellular longevity [81].

The isoprenoid pathway, which is essential for cholesterol metabolism, is a new target of modulating macrophage function. The use of statins has been associated with interstitial lung abnormalities in smoking individuals, a condition known to have a predominance of M2 macrophages [82]. Statins have potent anti-inflammatory properties and are known to orchestrate the immune response toward alternative activation *via* regulating isoprenoid biosynthesis [83]. The inhibition of farnesyltransferase, geranylgeranyltransferase I, and geranylgeranyltransferase II decreases cell survival, migration, and proliferation in many cancers [84]. Activation of Rac1 by geranylgeranylation in alveolar macrophages promotes characteristics of M2 macrophages and associates with the development of oxidative stress and pulmonary fibrosis. Digeranyl bisphosphonate (DGBP), which impairs geranylgeranylation of Rho GTPases by inhibiting geranylgeranyl diphosphate synthase, reduces mitochondrial oxidative stress and abrogates progression of pulmonary fibrosis by inhibiting Rac1 activation and its mitochondrial translocation [85].

Both the Akt pathway and the isoprenoid pathway are important in maintaining cell survival. Akt regulates apoptosis by modulating isoprenoid pathway. Akt-deficient macrophages (Akt^+/−^) have a significant increase of apoptosis. Akt overexpressing macrophages have a distinct M2 polarization pattern and promote fibrotic development. Conversely, Akt^+/−^ mice are protected from developing pulmonary fibrosis [86]. Statins activate Akt and, as previously mentioned, the use of statins has been associated with interstitial lung abnormalities in smoking individuals [82,87]. Surface scavenger receptors, which are crucial for internalization of extracellular oxidized lipid particles, are capable of regulating macrophage polarization. CD36 is known to be important for triacylglycerol substrate uptake and sequential oxidative phosphorylation, which leads to M2 polarization [63]. Another surface scavenger receptor, MARCO (macrophage receptor with collagenous structure) has been shown to increase mitochondrial oxidative stress and regulates macrophage polarization. Over-expression of wild-type MACRO leads to increased M2 gene expression, while knockdown of MARCO reduces M2 gene expression. Moreover, MACRO^−/−^ mice are protected from developing asbestos-induced pulmonary fibrosis. Inhibition of the scavenger receptor by fucoidan reduces mitochondrial H_2_O_2_ production, which inhibits macrophage M2 polarization [88]. Similarly, MARCO can limit inflammatory response as MARCO-deficient mice show an early-enhanced development of inflammation in response to influenza infection [89]. CD163, a scavenger receptor for the hemoglobin-haptoglobin complex, is expressed at high level by M2 macrophages in patients with idiopathic pulmonary fibrosis [90].

## Conclusion

Macrophage polarization is a dynamic process that our immune system utilizes to maintain an immunological homeostasis. Various factors influence polarization and further investigation for metabolic regulation in shaping the macrophage differential profile is warranted. In this review, we briefly summarize recent advances in macrophage polarization with special attention to their relevance to specific disease conditions and metabolic regulation of polarization. Understanding these metabolic switches can facilitate the development of targeted therapies for various diseases related to the distinct macrophage subtype.
